# Basic Psychological Needs, Passion, and Well-Being at Work: Evidence from Tunisian Physical Education Teachers

**DOI:** 10.3390/healthcare14091171

**Published:** 2026-04-27

**Authors:** Slim Saaidia, Hamdi Henchiri, Hela Znazen, Amr Chaabeni, Abdulazeem Alotaibi, Abdullah H. Alliheibi, Noureddine M. Ben Said, Fairouz Azaiez

**Affiliations:** 1 Higher Institute of Sport and Physical Education of Sfax, University of Sfax, Sfax 3029, Tunisia; slim331981@gmail.com (S.S.); fairouz.kyranis@yahoo.fr (F.A.); 2 Research Laboratory Education, Motricity, Sport Health EM2S, LR19JS01, High Institute of Sport and Physical Education of Sfax, University of Sfax, Sfax 3029, Tunisia; 3 Occupational Medicine and Professional Pathologies Service, Gafsa Regional Hospital, Gafsa 7100, Tunisia; 4 Department of Physical Sports Sciences, College of Sports Sciences and Physical Activity, Princess Nourah Bint Abdulrahman University, P.O. Box 84428, Riyadh 11671, Saudi Arabia; 5 Research Laboratory of Technology and Medical Imaging (LR12ES06), Center for Musculoskeletal Biomechanics Research, Faculty of Medicine, University of Monastir, Monastir 5000, Tunisia; amrch97@gmail.com; 6 Department of Physical Medicine and Rehabilitation, Faculty of Medicine, University of Monastir, Monastir 5000, Tunisia; 7 Department of Physical Education and Kinesiology, College of Education, Qassim University, Qassim 52571, Saudi Arabia; atiebya@qu.edu.sa; 8 Department of Physical Education, College of Sport Sciences and Physical Activity, King Saud University, Riyadh 11451, Saudi Arabia; aalliheibi@ksu.edu.sa; 9 Department of Biomechanics and Motor Behavior, College of Sport Sciences and Physical Activity, King Saud University, Riyadh 11451, Saudi Arabia; nbensaeed@ksu.edu.sa

**Keywords:** basic psychological needs, passion, psychological well-being, physical education teachers, Self-Determination Theory

## Abstract

**Background**: Grounded in Self-Determination Theory (SDT) and the Dualistic Model (DM) of Passion, this study examined the motivational mechanisms underlying psychological well-being among Tunisian physical education teachers. The objectives were twofold: to examine validity evidence of the Arabic version of the Basic Psychological Need Satisfaction and Frustration Scale (BPNSFS) and to test an integrative structural model linking harmonious passion, need satisfaction and frustration, well-being, vitality, happiness, and perceived stress. **Methods**: A representative sample of physical education teachers (1238) completed standardized instruments to assess passion, basic psychological needs, and well-being. To conduct exploratory and confirmatory factor analyses, the group was randomly divided into two independent subgroups. Reliability and validity were assessed using additional psychometric indicators, and a structural equation model was used to test the hypothesized relationships. **Results**: The results support the multidimensional structure and psychometric validity of the scale in the Tunisian context. Harmonious passion appears to be a positive factor in the satisfaction of psychological needs and a negative factor in cases of frustration. The satisfaction of these needs is closely linked to a high level of well-being, whereas their dissatisfaction is associated with adverse consequences. Well-being is also associated with increased vitality, greater happiness, and reduced stress, reflecting adaptive psychological functioning. **Conclusions**: Harmonious passion and basic psychological need satisfaction emerge as central resources for sustaining teacher well-being, vitality, and resilience against stress in educational contexts.

## 1. Introduction

In today’s professional environments, where psychosocial demands are increasing and performance is being sought, comprehending the motivational determinants of employee well-being and optimal functioning is a significant scientific and organizational challenge, a robust body of research identifies the satisfaction of basic psychological need autonomy, competence, and relatedness as a fundamental predictor of psychological well-being, vitality, and engagement, while also buffering against burnout and distress [1,2]. The Dualistic Model of Passion distinguishes between harmonious passion (HP) and obsessive passion (OP), which are both marked by a flexible and autonomous integration of work into identity [3]. HP is consistently linked to positive adaptation, whereas OP correlates with negative outcomes such as emotional exhaustion [4].

The teaching profession is widely recognized as emotionally demanding and psychologically taxing, frequently exposing educators to chronic stressors that may culminate in negative outcomes such as emotional exhaustion and burnout [4,5]. Within this context, understanding the motivational processes that sustain teachers’ well-being and vitality has become a central concern in contemporary educational psychology. Two influential theoretical frameworks have substantially contributed to this understanding: the Dualistic Model of Passion [5] and Self-Determination Theory [1,6]. Although both perspectives emphasize internal motivational dynamics and optimal functioning, their integration remains theoretically underdeveloped.

According to the Dualistic Model of Passion, individuals may internalize their professional activity in either a harmonious or obsessive manner [6]. Harmonious passion (HP) results from an autonomous internalization of the activity into one’s identity and is associated with flexibility, psychological well-being, and adaptive functioning. In contrast, obsessive passion (OP) stems from a controlled internalization, often leading to rigid persistence, conflict between life domains, and vulnerability to ill-being outcomes [3]. Empirical evidence indicates that harmonious passion is positively associated with work engagement, life satisfaction, and vitality, whereas obsessive passion is more strongly linked to stress and emotional exhaustion [7,8,9,10].

In parallel, Self-Determination Theory posits that optimal functioning depends on the satisfaction of three basic psychological needs: autonomy, competence, and relatedness [1,7]. When these needs are fulfilled, individuals experience enhanced intrinsic motivation, psychological well-being, and sustained vitality. Conversely, need frustration is associated with maladaptive outcomes, including burnout and diminished well-being [10]. The predictability of higher engagement and lower emotional exhaustion in occupational contexts is closely tied to teachers’ perceptions of autonomy support, professional competence, and collegial relatedness [11].

Despite their conceptual complementarity, these two influential frameworks are rarely integrated within a unified explanatory model. Specifically, it remains unclear whether passion and need satisfaction function as independent predictors of well-being, or whether need satisfaction constitutes the central psychological mechanism through which passion, particularly harmonious passion, translates into positive outcomes. From an Self-Determination Theory perspective, harmonious passion may foster well-being precisely because it promotes experiences of autonomy, competence, and relatedness within the professional role. However, empirical investigations explicitly testing this mediational pathway remain limited.

This theoretical gap is especially salient in complex professional environments where motivational resources are continuously challenged. The teaching of physical education (PE) in Tunisia represents one such context. Tunisian PE teachers often experience a paradoxical professional reality: strong personal commitment to sport and education coexists with structural constraints such as limited material resources, institutional pressures, overcrowded classes, and ambiguous societal valuation of the discipline. These contextual stressors may threaten basic psychological need satisfaction, thereby increasing vulnerability to emotional exhaustion. At the same time, PE teaching is frequently chosen out of genuine passion for sport and youth development, making it an ideal natural laboratory for examining how different forms of passion operate under constraint.

From this perspective, the present study aims to test, using structural equation modeling, a comprehensive motivational model that links passion, the fulfillment of basic psychological needs, and indicators of well-being among physical education teachers in Tunisia. Based on the theoretical frameworks employed, the following hypotheses are proposed:

**H1.**  *Harmonious passion and the fulfillment of basic psychological needs will be positively related to mental well-being and subjective vitality, whereas obsessive passion will be negatively related or show insignificant relationships with these indicators.*

**H2.**  *Harmonious passion will be positively related to the fulfillment of basic psychological needs, whereas obsessive passion will be positively related to the frustration of these same needs.*

**H3.**  *The fulfillment of basic psychological needs will play an important mediating role in the relationship between harmonious passion and indicators of well-being, thereby constituting a closely related motivational process that explains optimal engagement and vitality.*

By examining these relationships in a non-Western, resource-constrained educational context, this research offers distinct theoretical and practical contributions. Theoretically, it advances motivational science by integrating the Dualistic Model of Passion within a Self-Determination Theory framework, clarifying the psychological processes underlying optimal functioning in education. Practically, it identifies actionable levers for educational policy and school leadership, namely, fostering autonomy-supportive environments and cultivating harmonious passion to enhance teacher sustainability, well-being, and professional effectiveness within the Tunisian educational system and comparable contexts.

## 2. Materials and Methods

### 2.1. Participants

Given that the validation of a scale is a continuous and gradual process based on the accumulation of evidence from various sources [1,12], this study aims to assess the psychometric properties and examine validity evidence of the validity and reliability of the Basic Psychological Need Satisfaction and Frustration Scale (BPNSFS-24) in thecontext of physical education in Tunisia. To this end, a sample of 1238 teachers from various subjects was assembled, including 578 men and 651 women, selected from five districts in Tunisia (Carthage, Medjerda, Sahel, Atlas, and Ksour). To verify the reliability of the translated BPNSFS-24 version, 50 participants were selected for a test–retest procedure. Participants were recruited via convenience sampling from various schools during the 2024–2025 academic year. The survey involved in-service teachers aged between 24 and 60 years, with at least one year of experience in their field. Teachers on extended leave were excluded at the time of the survey. Questionnaires that were incomplete, contained duplicate responses, or were completed too quickly were considered invalid. Participation was entirely voluntary, and data confidentiality was strictly maintained throughout the study.

### 2.2. Measures

Passion Scale (PS-6), adapted for work. Vallerand and colleagues’ (2003) [6] Passion Scale was used to assess participants’ harmonious passion for their work. For this research, only the six-item subscale measuring harmonious passion was implemented. A sample item is “My work is in harmony with other activities in my life.” The Passion Scale has demonstrated high levels of validity and reliability across several life domains, including work [6,9,13,14]. Items were rated on a 7-point Likert scale, ranging from (1) strongly disagree to (7) strongly agree.

Subjective Vitality Scale (SVS-7). The 7-item self-report instrument developed by Ryan and Frederick (1997) [15] was used. It assesses an individual’s perceived energy and aliveness. Responses are typically collected on a 7-point Likert scale (from 1 = not at all true to 7 = very true). A high score indicates significant psychological vitality, whereas a low score reflects a state of fatigue or energy deficit. The scale possesses strong psychometric properties and has shown high internal consistency across various research contexts [12,16].

Basic Psychological Need Satisfaction and Frustration Scale (BPNSFS-24). This scale examines both the satisfaction and frustration related to the three core psychological needs: autonomy, competence, and relatedness. It allows for a precise differentiation between positive and negative experiences of the motivational context, in line with Self-Determination Theory. Participants indicated their agreement on a 5-point Likert scale ranging from 1 (completely false) to 5 (completely true). The scale has excellent psychometric properties and has been validated in diverse cultural and professional contexts [15].

**Psychological Well-Being Scale (PWB-18).** Developed by Ryff (1989) [17], this model assesses positive psychological well-being across six dimensions: autonomy, environmental mastery, personal growth, positive relations with others, purpose in life, and self-acceptance. Items are rated on a 7-point Likert-type scale (from 1 = strongly disagree to 7 = strongly agree), yielding dimensional scores or a global well-being score. This scale adopts a eudaimonic perspective on well-being, focused on the realization of personal potential. It demonstrates a robust factor structure, good internal consistency, and satisfactory convergent and discriminant validity [18,19].

Perceived Stress Scale (PSS-10). This scale determines the degree of stress perceived in a professional context, considering work demands, time pressure, and organizational constraints. Items are typically rated on a 5-point Likert-type scale (e.g., from 1 = never to 5 = very often), with a high score indicating a high level of perceived professional stress. This scale is widely used due to its high validity and reliability in work-related stress research [17].

Subjective Happiness Scale (SHS-4). Developed by Lyubomirsky and Lepper (1999) [20], it assesses an individual’s overall level of subjective happiness. It consists of 4 items rated on a 7-point Likert scale (from 1 = not at all to 7 = extremely), yielding an average happiness score. The scale has good psychometric properties and has demonstrated validity across different cultural environments [21].

### 2.3. Procedure

The adaptation procedure followed strict translation and validation protocols [22]. As recommended, two to three separate translations were performed by two panels of competent translators with excellent command of the target language, prioritizing their native tongue. One panel was briefed on the questionnaire’s objectives, while the other was deliberately kept unaware to ensure a translation accurate to the target population’s language.

To enhance methodological rigor and minimize biases associated with random sampling, we conducted an exploratory factor analysis (EFA) and a confirmatory factor analysis (CFA) on two distinct subgroups. In addition, a varimax (orthogonal) rotation was applied during the exploratory factor analysis, assuming the independence of the factors and thus providing a clearer, more stable, and more interpretable factor structure, in accordance with psychometric recommendations.

The initial principal component analysis (PCA), performed on the first sample of 619 teachers, allowed for the examination of the underlying factor structure and the identification of suitable items. Subsequently, a CFA was performed on a second independent sample of 619 teachers and psychometric properties to test the factor model derived from the EFA using standard fit indices. This two-cycle approach enhances the structural robustness and psychometric reliability of the instrument [20,23,24].

### 2.4. Statistical Analysis

For the exploratory factor analysis (EFA), this research employed principal component analysis to extract factors with eigenvalues greater than 1. An oblique rotation (varimax) was implemented for factor rotation, which demonstrated that certain correlations between factors were consistent with theory. For the confirmatory factor analysis (CFA), we examined common method variance (CMV) and the measurement invariance of the model. The most recent attempt involved creating a higher-order model based on the first-order factor model. All analyses were performed using SPSS 26.0 and AMOS 24.0.

## 3. Results

### 3.1. Sociodemographic Characteristics

In factor analysis, it is recommended that researchers strive to obtain samples of 300 or more participants to enhance confidence in the results [24,25]. Accordingly, a sample of 1238 teachers from various disciplines was selected for the purpose of validating the Basic Psychological Need Satisfaction and Frustration Scale.

Our study population consisted of 1238 teachers, with a slight predominance of female participants (52.6% women vs. 47.4% men). Specifically, the sample included 587 men and 651 women teaching in different regions of Tunisia (at the middle school or high school level).

**Figure 1:**
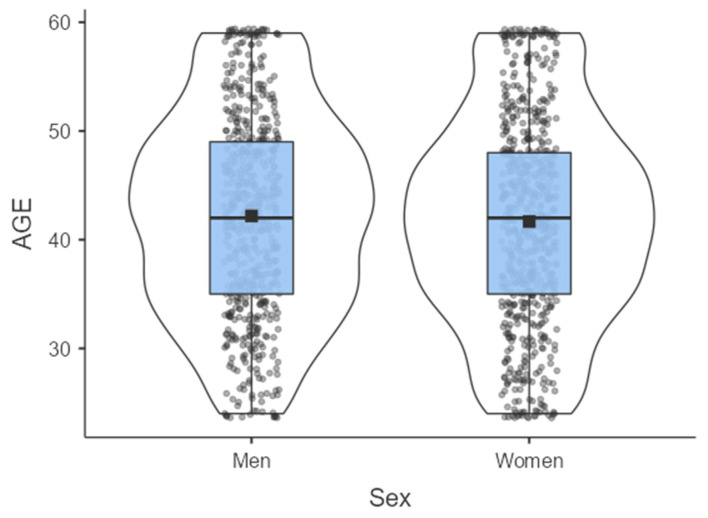
Population distribution by age and sex.

Participation was on a voluntary basis, with informed consent guaranteed and data confidentiality protected. To maintain data integrity, questionnaires that were incomplete, were completed too quickly, or contained duplicate responses were excluded. Participant age ranged from 24 to 59 years (M = 41.9, SD = ±9.42), with a mean age of 42.2 (SD = ±9.37) for male teachers and 41.7 (SD = ±9.49) for female teachers. Participants resided in rural (58.5%) and urban (51.5%) areas, reflecting geographical diversity. The regions of Carthage and Sahel were the most represented, indicating good territorial coverage. The majority of teachers were married (33.9%), suggesting a predominantly stable family life. They primarily taught physical education (37.6%), followed by languages and sciences. Nearly 30% had over 20 years of seniority. Finally, the majority of participants had 1 to 2 children (48.47%) or 3 to 4 children (33.68%), while 17.85% had no children, reflecting a population largely engaged in family responsibilities (see Table 1). The normality index (the data are distributed according to a normal law, with Skewness (0.011) and Kurtosis (−0.785) tending toward “zero”) indicates that the population follows a normal distribution (Figure 1). The observed skewness and kurtosis values fall within the generally accepted thresholds for distribution normality [26]. This permits the use of parametric analyses and structural equation modeling without specific adjustments.

### 3.2. Exploratory Factor Analysis

To assess the psychometric quality of the construct, our questionnaire was subjected to orthogonal (varimax) factor analysis based on its 24 items [15]. To simplify the table content, factor loadings are denoted by 0.3, in accordance with the criteria selected by [27,28]. The data demonstrate that the Basic Psychological Need Satisfaction and Frustration Scale [15] is distinguished by very high internal consistency (alpha = 0.915) and remarkable stability over time (test–retest *r* = 0.903).

**Figure 2:**
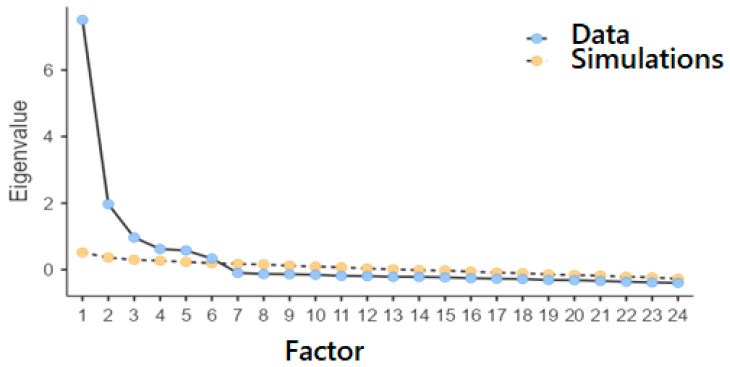
Scree plot of the Basic Psychological Need Satisfaction and Frustration Scale.

The first step in the analysis involved conducting an exploratory factor analysis (EFA) to examine the underlying structure of the Arabic version of the BPNSFS. As part of a preliminary exploratory approach, we used principal component analysis (PCA) to identify potential structures and examine the arrangement of the items [29]. A varimax (orthogonal) rotation was applied, which means that the extracted factors are assumed to be independent of one another. Nevertheless, due to the theoretical link between the concept’s dimensions, the results were interpreted with caution and supplemented by confirmatory factor analysis (CFA). The suitability of the data for factor analysis was confirmed by the Kaiser–Meyer–Olkin index (KMO = 0.929), which was well above the recommended threshold, as well as by Bartlett’s sphericity test (*p* < 0.001), attesting to the data’s suitability for factor extraction [30]. Bartlett’s test of sphericity yielded a significant result (χ^2^ = 6702.873, df = 276, *p* < 0.001), indicating that the correlations are sufficiently strong for factor determination [31]. We analyzed a scree plot (Figure 2) to determine the optimal number of factors to extract. The plot’s intent was to identify factors with eigenvalues greater than 1. The parallel analysis, conducted with 619 random data points, highlighted a six-dimensional solution, corresponding to the dimensions of autonomy satisfaction, autonomy frustration, relatedness satisfaction, relatedness frustration, competence satisfaction, and competence frustration. These six factors explained 34.088%, 11.193%, 6.869%, 5.550%, 5.142%, and 4.248% of the total variance, respectively, thereby confirming the multidimensional structure of the Basic Psychological Need Satisfaction and Frustration Scale.

The assessment of the internal consistency of the Basic Psychological Need Satisfaction and Frustration Scale dimensions was performed using Cronbach’s alpha (α) and McDonald’s omega (ω) coefficients to improve the assessment of consistency [32,33,34]. The results show Cronbach’s alpha values ranging from 0.822 to 0.845, and McDonald’s omega coefficients ranging from 0.829 to 0.838, indicating good internal consistency of the scales. These values substantially exceed the recommended threshold of 0.70, indicating that the items reliably measure the intended latent constructs.

- Factor Structure and Variance Explained

The designed model encompasses six major factors that explain 67.479% of the total variance. The first component, with an eigenvalue of 8.181, groups items associated with autonomy satisfaction (items 1–4). The second component, with an eigenvalue of 2.686, groups items associated with autonomy frustration (items 5–8). The third component, with an eigenvalue of 1.649, groups items related to relatedness satisfaction (items 9–12). The fourth component, with an eigenvalue of 1.332, groups items associated with relatedness frustration (items 13–16). The fifth component, with an eigenvalue of 1.234, groups items associated with competence satisfaction (items 17–20). Finally, the sixth component, with an eigenvalue of 1.019, groups items associated with competence frustration (items 21–24). Following the examination of eigenvalues, Figure 2 illustrates the factor loadings for the BPNSFS components on each of the identified factors.

Each component of the Basic Psychological Need Satisfaction and Frustration Scale demonstrated a strong relationship with its associated factor, reinforcing the six-factor structure of the Arabic-BPNSFS model. The examination of uniqueness indices (see Table 2) shows that the majority of items present values below 0.40, illustrating appropriate representation by the chosen factorial solution. Consequently, the corresponding communality values (1—uniqueness), ranging approximately from 0.46 to 0.63, attest to a good representation of the items by the extracted factors [30,35]. Overall, these results support the validity and coherence of the questionnaire’s factor structure.

- Composite Reliability and Average Variance Extracted

The observed factor loadings all range from moderate to high (≈0.70 to 0.79) (see Table 2), exceeding the suggested minimum threshold of 0.50, which confirms good convergent validity at the item level [35]. Although for some dimensions the AVE is slightly below the strict threshold of 0.50, these values remain acceptable within the framework of exploratory or cross-cultural validation, provided the factor loadings are high and homogeneous [36]. According to Hair et al. [30], an AVE below 0.50 can be acceptable if the Composite Reliability (CR) is adequate, which is often the case when items make a significant contribution to the latent construct. The results therefore suggest that the measured dimensions show satisfactory internal consistency and capture a significant portion of the construct’s variance, supporting the overall convergent validity of the scale. These findings are in accordance with methodological guidelines for factor analysis in psychological and social sciences [37].

- Sensitivity Analysis

The analysis reveals that basic psychological need satisfaction presents a moderate to strong association with the examined variables, displaying significant effect sizes for most relationships (η^2^ = 0.139 to 0.310). The most significant correlations are established with happiness (η^2^ = 0.310), well-being (η^2^ = 0.254), and harmonious passion (η^2^ = 0.221), highlighting their primary importance in the fulfillment of psychological needs. In contrast, age, place of residence, vitality, and stress have a moderate but significant impact on basic psychological need satisfaction.

Conversely, association analyses show moderate to strong effects between basic psychological need frustration and all the examined variables (η^2^ = 0.355 to 0.501). The most significant effects are observed in the domains of well-being, harmonious passion, and vitality, indicating that these factors account for a considerable portion of the variation in need frustration.

- Convergent and Discriminant Validity

The observed correlations clearly confirm the convergent validity of the examined constructs (Table 3). Psychological need satisfaction is significantly and positively correlated with harmonious passion (*r* = 0.297), well-being (*r* = 0.367), happiness (*r* = 0.456), and vitality (*r* = 0.215), while being negatively correlated with stress (*r* = −0.124). Similarly, harmonious passion demonstrates substantial correlations with well-being (*r* = 0.518), happiness (*r* = 0.599), and vitality (*r* = 0.563), alongside a significant negative correlation with stress (*r* = −0.442). This indicates strong theoretical convergence among these positive variables. Conversely, need frustration shows a positive association with stress (*r* = 0.260) and a negative link with well-being indicators.

### 3.3. Confirmatory Factor Analysis

**Figure 3:**
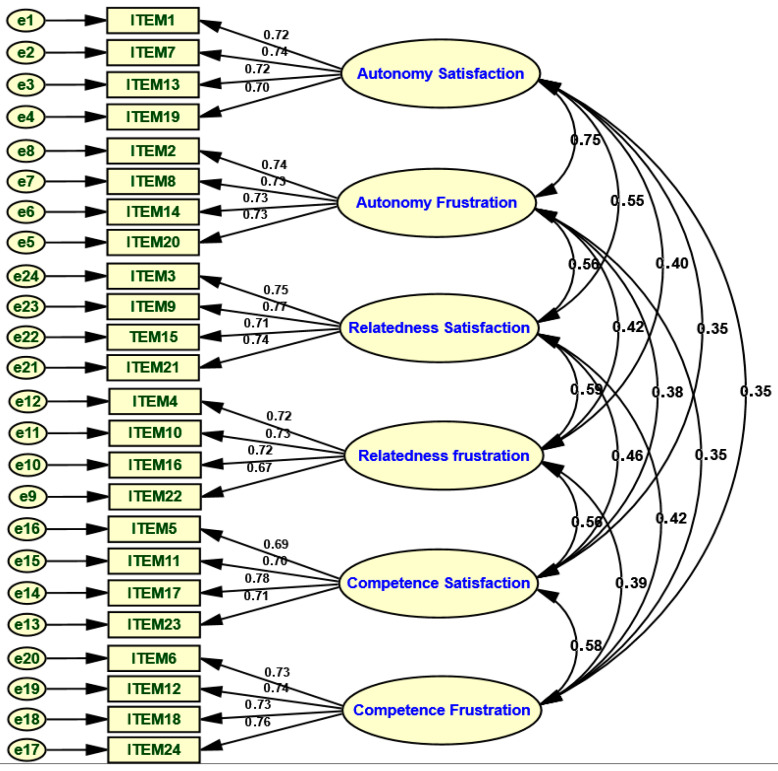
Standardized regression loadings of single-factor CFA.

The fit indices demonstrate an almost perfect, indeed excellent, adaptation of the model to the empirical data (see Table 4 and Figure 3). The χ^2^ statistic (245.49), referenced against 237 degrees of freedom, is low, and the χ^2^/df ratio = 1.03 is well below the recommended threshold values (<2 or <3), indicating an excellent overall model fit (Kline, 2023 [26]). The absolute fit indices (GFI = 0.968; AGFI = 0.960) considerably surpass the 0.90 threshold, suggesting the model faithfully reflects the observed covariance matrix. The incremental fit indices corroborate this precision of fit, with very high values for NFI (0.960), CFI (0.999), IFI (0.999), and TLI (0.998), all above the strict 0.95 benchmark recommended for well-specified models [38]. The residual indicators are also very good, as shown by the small values of RMR (0.011) and SRMR (0.022), well below the 0.08 threshold, which indicate minimal residual differences between the observed and estimated covariances [37]. Finally, the very low RMSEA (0.008) reveals a near-absence of approximation error and attests to the model’s robustness, parsimony, and stability, even when considering its complexity and the sample size [38,39].

### 3.4. Structural Equation Modeling

**Figure 4:**
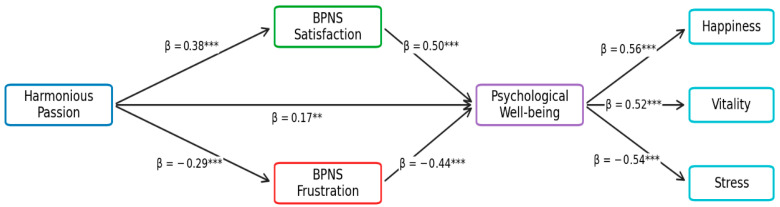
The impact of passion and basic need satisfaction on psychological well-being and vitality at work: the case of physical education teachers. Note: *p* < 0.01 (**), *p* < 0.001 (***).

In terms of the effect of passion and basic need satisfaction on psychological well-being and vitality at work for the case of physical education teachers, the structural equation model (Figure 4) highlights the paramount importance of harmonious passion and the satisfaction/frustration of basic psychological needs (BPNs) for understanding the psychological well-being of physical education teachers, which plays a crucial mediating role in their impacts on happiness, vitality, and stress.

Harmonious passion has a significant positive impact on psychological need satisfaction (β = 0.38, *p* < 0.001) (Figure 4), supporting the premise that an autodetermined engagement fosters autonomy, competence, and relatedness. Conversely, it is negatively associated with psychological need frustration (β = −0.29, *p* < 0.001), confirming a protective role against experiences of pressure, constraint, or devaluation. Furthermore, harmonious passion also appears to have a direct positive effect on psychological well-being (β = 0.17, *p* < 0.01), highlighting that its benefits are not solely mediated by psychological needs but also stem from specific affective and motivational mechanisms.

Psychological need satisfaction emerges as an important predictor of well-being (β = 0.50, *p* < 0.001) (Figure 4), attesting to the fundamental principles of Self-Determination Theory. In contrast, need frustration has a significant negative impact on well-being (β = −0.44, *p* < 0.001), underscoring that the deficit in satisfaction is not merely a lack of resources but a key determinant of psychological vulnerability. These findings support the concept that need satisfaction and frustration are two separate mechanisms with asymmetric consequences for psychological functioning.

In the model, psychological well-being plays a balancing role, channeling its effects toward subjective health and energetic indicators. It is strongly linked to happiness (β = 0.56, *p* < 0.001) and vitality (β = 0.52, *p* < 0.001) (Figure 4), indicating that optimal psychological functioning leads to a positive emotional state and a high level of psychic energy. Conversely, psychological well-being is negatively related to stress (β = −0.54, *p* < 0.001), attesting to its preventive effect against psychological tensions and strains.

## 4. Discussion

Theoretical implication

The present study aimed to examine the psychometric validity of the Arabic version of the Basic Psychological Need Satisfaction and Frustration Scale (BPNSFS) and to test an integrative motivational model linking harmonious passion, basic psychological needs, psychological well-being, vitality, happiness, and stress among Tunisian physical education teachers. The findings provide strong empirical support for the six-factor structure of the BPNSFS and confirm the central role of basic psychological need satisfaction and frustration as distinct yet complementary motivational processes. Moreover, the structural model highlights harmonious passion as a key antecedent of need satisfaction and psychological well-being, which in turn serves as a critical mediator toward both positive (happiness and vitality) and negative (stress) psychological outcomes.

These results contribute to the growing body of research grounded in Self-Determination Theory [7] and the Dualistic Model of Passion [3], offering novel evidence from a non-Western, resource-constrained educational context.

In our study, we did see that the six-dimensional structure of the BPNSFS, which is related to autonomy, competence, and relatedness satisfaction and frustration put forth by other researchers, did in fact play out. Also, we saw very good fit indices in the confirmatory factor analysis (CFI = 0.999; RMSEA = 0.008), which in turn supports the theory put forth by [19], that this model is very much applicable to Tunisian teachers. Also, we see that what we report is in agreement with past validations which have been done in very diverse cultural settings, which include Europe, Asia, and Latin America [15,40,41]. We also report that we supported the distinction between need satisfaction and need frustration, which is a key point in Self-Determination Theory put forth [11,15,42], frustration is a separate entity from the lack of satisfaction, which in turn has bad outcomes. This is very present in occupational settings like teaching, which has issues of institutional constraints, work load, and low autonomy, which in turn go after psychological needs. Also, a main result of our study is that we saw a very strong positive relationship between what we term harmonious passion and basic psychological need satisfaction. Also, we saw that it has a negative association with need frustration.

One of the central findings of this study is the strong positive relationship between harmonious passion and basic psychological need satisfaction, alongside its negative association with need frustration. These results align with the Dualistic Model of Passion, which posits that harmonious passion reflects an autonomous internalization of work activities, allowing individuals to engage flexibly and coherently with other life domains [3,8].

In line with previous studies conducted among teachers and professionals [9,14,43], harmonious passion emerged as a powerful motivational driver that supports autonomy, competence, and relatedness. In the specific context of physical education teaching, where professional identity is often strongly tied to personal values and embodied practice, harmonious passion may function as a protective psychological resource that buffers against institutional pressures and role overload.

Consistent with Self-Determination Theory, basic psychological need satisfaction was a strong positive predictor of psychological well-being, whereas need frustration exerted a substantial negative effect. These findings corroborate a large body of the literature demonstrating that need satisfaction fosters optimal functioning, vitality, and mental health, while need frustration predicts burnout, distress, and ill-being [2,10,12].

Notably, the magnitude of the negative effect of need frustration on well-being was comparable to, or even stronger than, the positive effect of need satisfaction. This asymmetry supports theoretical claims that need-thwarting experiences may have particularly detrimental consequences, especially in high-demand professions such as teaching [11]. For physical education teachers operating under structural constraints (e.g., limited resources, large class sizes, and low institutional recognition), persistent need frustration may undermine long-term professional sustainability.

A major contribution of this study lies in the identification of psychological well-being as a central mediating mechanism linking motivational processes to both positive and negative outcomes. Psychological well-being strongly predicted happiness and vitality, while exhibiting a robust negative association with perceived stress. This pattern is consistent with Ryff’s eudaimonic model, which emphasizes meaning, personal growth, and environmental mastery as core components of optimal functioning [18,19].

The mediating role of well-being supports integrative motivational frameworks suggesting that need satisfaction and passion influence distal outcomes primarily through their impact on individuals’ overall psychological functioning [42,44]. In this sense, well-being acts as a psychological “hub” that channels motivational energy into sustained vitality and resilience while simultaneously reducing vulnerability to stress.

Implications for Stress, Vitality, and Happiness

The strong negative association between psychological well-being and stress underscores the protective role of motivational resources in occupational health. These findings are in line with stress models emphasizing the importance of internal resources in coping with job demands [45,46,47]. Teachers who experience high need satisfaction and harmonious passion are likely to appraise stressors as more manageable, thereby reducing their perceived impact.

Furthermore, the strong links between well-being, vitality, and happiness confirm that Self-Determination Theory-based constructs are closely aligned with both hedonic and eudaimonic indicators of mental health [12,21]. Vitality, in particular, represents a core experiential outcome of need satisfaction and autonomous motivation, reflecting not merely the absence of fatigue but also the presence of psychological energy [7].

From a practical standpoint, the findings suggest that interventions aimed at enhancing teacher well-being should prioritize the satisfaction of basic psychological needs and the cultivation of harmonious passion. Educational policies and school leadership practices that support autonomy (e.g., pedagogical freedom), competence (e.g., professional development), and relatedness (e.g., collegial support) are likely to foster sustainable motivation and reduce stress.

In the Tunisian context, where structural constraints are often unavoidable, even small improvements in the motivational climate may yield substantial benefits. Training programs for school administrators could integrate Self-Determination Theory principles to promote need-supportive leadership, thereby enhancing both teacher well-being and educational quality.

Limitations and Future Research Directions

Several limitations of this study should be noted. First, its cross-sectional design does not allow for the establishment of causal relationships between the variables examined. Second, the applicability of the findings is limited to comparable educational settings, particularly within the Tunisian context. Third, the use of self-reported measures may introduce social desirability bias.

To better establish cause-and-effect relationships, future studies should focus on longitudinal or experimental designs. It would also be advisable to incorporate contextual and organizational factors (such as school climate and leadership) and to test multilevel and cross-cultural models to enhance the robustness and generalizability of the findings.

Future research should extend this model to other educational and professional populations and explore potential moderators such as gender, career stage, or organizational climate. Additionally, the inclusion of obsessive passion in future models would provide a more comprehensive test of the dualistic passion framework.

## 5. Conclusions

In conclusion, this study provides robust psychometric and theoretical evidence supporting the validity of the BPNSFS and the central role of basic psychological needs and harmonious passion in promoting psychological well-being among physical education teachers. The findings support the assumptions of Self-Determination Theory, demonstrating that the fulfillment and unfulfillment of basic psychological needs are distinct processes that affect well-being, while also supporting the Dualistic Model of Passion through the differentiated impacts of harmonious passion and obsessive passion. The mediating role of psychological needs highlights a related motivational process that explains engagement and optimal functioning. It should be noted that the indirect effects were estimated using the bootstrapping technique (with a 95% confidence interval), a method that allows for the assessment of the significance of mediating effects without assuming normal distributions. The confidence intervals obtained confirm the robustness and credibility of the observed mediating effects. From an applied perspective, these results underscore the need for educational leadership that fosters autonomy, competence, and interpersonal relationships through actions such as involvement in decision-making, professional development, and the improvement of the collaborative climate. With regard to educational policies, incorporating concepts from Self-Determination Theory into the training of teachers and educational administrators appears essential for sustainably optimizing well-being and the quality of education. In the Tunisian context, characterized by structural constraints, limited resources, and rising professional demands, these findings take on particular significance. They suggest that improving the motivational climate is a realistic and effective way to promote teachers’ well-being, even without major structural reforms. In practice, this means strengthening teaching methods that encourage autonomy, developing professional development programs focused on a sense of competence, and fostering collaborative environments that support interpersonal relationships. Future longitudinal, experimental, and cross-cultural studies incorporating additional variables such as emotional regulation or organizational climate are needed to evaluate more sophisticated, multi-level models.

## Figures and Tables

**Table 1:** Characteristics by sex.

Sex		Men		Women		Total	
		Frequency	%	Frequency	%	Frequency	%
		578	47.42	651	52.58	1238	100
Age		$578\;\bar{x}$ = 42.2 (σ ± 9.37)		$651\;\bar{x}$ = 41.7 (σ ± 9.49)		$1238\;\bar{x}$ = 41.9 (σ ± 9.42)	
Residence	Urban	282	28.9	355	28.7	637	51.5
	Rural	305	24.6	296	23.9	601	58.5
Housing district	Carthage	211	17.0	204	16.5	415	33.52
	Medjerda	64	5.2	80	6.5	144	11.63
	Sahel	176	14.2	218	17.6	394	31.83
	Atlas	77	6.2	76	6.15.9	153	12.36
	Ksour	59	4.8	73	5.9	132	10.66
Civil status	Single	91	7.4	104	8.4	196	15.8
	Married	422	34.1	494	39.9	915	33.9
	Divorced	50	4.0	37	3.0	87	7.0
	Widowed	24	1.9	16	1.3	40	3.2
Specialty	Football	257	20.76	26	2.10	283	22.86
	Handball	121	9.77	102	8.24	223	18.01
	Basketball	55	4.44	64	5.17	119	9.61
	Volleyball	30	2.42	58	4.68	88	7.11
	Athletics	21	1.70	30	2.42	51	4.12
	Gymnastics	28	2.26	91	7.35	119	9.61
	Swimming	29	2.34	87	7.03	116	9.37
	Aerobics	32	2.58	175	14.14	207	16.72
	Other	14	1.13	18	1.45	32	2.58
Seniority	Less than 5 years	83	6.7	100	8.1	183	14.8
	5 to 10 years	114	9.2	129	10.4	243	19.6
	11 to 15 years	99	8.0	134	10.8	233	18.8
	16 to 20 years	95	7.7	112	9.0	207	16.7
	More than 20 years	196	15.8	176	14.2	372	30.0
Children	No Children	105	8.5	116	9.4	221	17.85
	1 to 2 Children	283	22.86	317	25.6	600	48.47
	3 to 4 Children	199	16.06	218	17.6	417	33.68

**Table 2:** Component matrix after varimax orthogonal rotation of the Basic Psychological Need Satisfaction and Frustration Scale.

Items		Factor Charges						AVE
		1	2	3	4	5	6	
1	I feel a sense of choice and freedom in the things I undertake	0.734						0.454
	*أشعر بأن لدي إحساسًا بالاختيار والحرية في الاشياء التي أقوم بها*							
2	I feel that my decisions reflect what I really want	0.756						0.406
	*أشعر أن قراراتي تعكس ما أريده حقًا*							
3	I feel my choices express who I really am	0.794						0.411
	*أشعر أن اختياراتي تعبّر عن هويتي الحقيقية*							
4	I feel I have been doing what really interests me	0.742						0.389
	*أشعر أنني أمارس ما يهمّني ويثير اهتمامي حقًا*							
5	Most of the things I do feel like “I have to”		0.753					0.393
	*أشعر أن معظم الأشياء التي أقوم بها تبدو وكأنها واجب مفروض عليّ*							
6	I feel forced to do many things I wouldn’t choose to do		0.778					0.482
	*أشعر بأنني مجبر على القيام بأشياء كثيرة لم أختر القيام بها*							
7	I feel pressured to do too many things		0.765					0.408
	*أشعر بالضغط للقيام بأشياء كثيرة*							
8	My daily activities feel like a chain of obligations		0.784					0.440
	*أشعر أن أنشطتي اليومية عبارة عن سلسلة من الالتزامات المفروضة*							
9	I feel that the people I care about also care about me			0.763				0.403
	*أشعر بأن الأشخاص الذين أهتمّ بهم يهتمّون بي أيضًا*							
10	I feel connected with people who care for me, and for whom I care			0.703				0.463
	*أشعر بارتباطي بالأشخاص الذين يهتمّون بي والذين أهتمّ بهم*							
11	I feel close and connected with other people who are important to me			0.761				0.446
	*أشعر بالقرب والتواصل مع أشخاص آخرين مهمّين في حياتي*							
12	I experience a warm feeling with the people I spend time with			0.749				0.472
	*أشعر بدفءٍ عاطفي مع الأشخاص الذين أقضي معهم وقتي*							
13	I feel excluded from the group I want to belong to				0.759			0.409
	*أشعر بأنني مُستبعَد من المجموعة التي أرغب في الانتماء إليها*							
14	I feel that people who are important to me are cold and distant towards me				0.726			0.372
	*أشعر بأن الأشخاص المهمّين بالنسبة لي يتعاملون معي ببرود وجفاء*							
15	I have the impression that people I spend time with dislike me				0.775			0.437
	*لديّ انطباع بأن الأشخاص الذين أقضي معهم وقتي لا يحبّونني*							
16	I feel the relationships I have are just superficial				0.712			0.538
	*أشعر بأن العلاقات التي أمتلكها سطحية في غالبتها*							
17	I feel confident that I can do things well					0.758		0.506
	*أشعر بالثقة في قدرتي على إنجاز الأمور بشكل جيّد*							
18	I feel capable at what I do					0.747		0.472
	*أشعر بأنني قادر على أداء ما أقوم به بكفاءة*							
19	I feel competent to achieve my goals					0.758		0.439
	*أشعر بأن لديّ الكفاءة اللازمة لتحقيق أهدافي*							
20	I feel I can successfully complete difficult tasks					0.745		0.419
	*أشعر بأنني أستطيع إتمام المهام الصعبة بنجاح*							
21	I have serious doubts about whether I can do things well						0.767	0.437
	*لديّ شكوك جدّية حول قدرتي على إنجاز الأمور بشكل جيّد*							
22	I feel disappointed with many of my performance						0.730	0.466
	*أشعر بخيبة أمل تجاه العديد من جوانب أدائي*							
23	I feel insecure about my abilities						0.724	0.445
	*أشعر بعدم الأمان بشأن قدراتي*							
24	I feel like a failure because of the mistakes I make						0.727	0.414
	*أشعر وكأنني فاشل بسبب الأخطاء التي أرتكبه*							

Extraction method: principal component analysis. Rotation method: varimax with Kaiser normalization. AVE = Average Variance Extracted.

**Table 3:** Convergent and discriminant validity.

Correlation							
	BPN.Sat	BPN.Fru	PASSION.H	WELL-BEING	HAPPINESS	VITALITY	STRESS
BPN.Sat	-						
BPN.Fru	0.435 **	-					
PASSION.H	0.297 **	0.374 **	-				
WELL-BEING	0.367 **	0.432 **	0.518 **	-			
HAPPINESS	0.456 **	0.281 **	0.599 **	0.668 **	-		
VITALITY	0.215 **	0.247 **	0.563 **	0.603 **	0.548 **	-	
STRESS	−0.124 **	−0.260 **	−0.442 **	−0.478 **	−0.387 **	−0.371 **	1

Note: BPN.Sat: basic psychological need satisfaction; BPN.Fru: basic psychological need frustration; PASSION.H: harmonious passion. **. The correlation is significant at the 0.01 level (two-tailed).

**Table 4:** Confirmatory factor analysis of the Basic Psychological Need Satisfaction and Frustration Scale.

Indices	χ^2^	df	χ^2^/df	GFI	NFI	AGFI	RMR	CFI	SRMR	IFI	TLI	RMSEA
Model	245.49	237	1.03	0.968	0.960	0.960	0.011	0.999	0.022	0.999	0.998	0.008

Note: N = 916, χ^2^: Chi-Square, df: Degree of Freedom, GFI: Goodness of Fit Index, NFI: Normed-Fit Index, AGFI: Adjusted Goodness-of-Fit Index, SRMR: Standardized Root Mean Square Residual, CFI: Comparative Fit Index, PNFI: Parsimony Normal Fit Index, RMSEA: Root Mean Square Error of Approximation.

## Data Availability

The data presented in this study are available from the corresponding author on request due to privacy and ethical restrictions, as the dataset contains sensitive information related to participants’ well-being at work.
